# Phytochemical Characterization, Antioxidant and Anti-Proliferative Properties of *Rubia cordifolia* L. Extracts Prepared with Improved Extraction Conditions

**DOI:** 10.3390/antiox11051006

**Published:** 2022-05-20

**Authors:** Ravikiran B. Humbare, Joyita Sarkar, Anjali A. Kulkarni, Mugdha G. Juwale, Sushil H. Deshmukh, Dinesh Amalnerkar, Manohar Chaskar, Maria C. Albertini, Marco B. L. Rocchi, Swapnil C. Kamble, Seeram Ramakrishna

**Affiliations:** 1 Department of Technology, Savitribai Phule Pune University, Ganeshkhind, Pune 411007, India; ravikiranhumbare007@gmail.com (R.B.H.); dpa54@yahoo.co.in (D.A.); manoharchaskar@gmail.com (M.C.); 2 Institute of Chemical Technology Mumbai, Marathwada Campus, Jalna, BT-6/7, Biotechnology Park, Additional MIDC Area, Aurangabad Road, Jalna 431203, India; j.sarkar@marj.ictmumbai.edu.in; 3 Department of Botany, Savitribai Phule Pune University, Ganeshkhind, Pune 411007, India; anjali.uop@gmail.com (A.A.K.); mugdhajuwale22@gmail.com (M.G.J.); 4 Maharashtra Arogya Kendra’s Sane Guruji Arogya Kendra, Pune 411028, India; sushildeshmukh@hotmail.com; 5 Department of Biomolecular Sciences, University of Urbino Carlo Bo, 61029 Urbino, Italy; maria.albertini@uniurb.it (M.C.A.); marco.rocchi@uniurb.it (M.B.L.R.); 6 Center for Nanofibers and Nanotechnology, Department of Mechanical Engineering, National University of Singapore, Singapore 119260, Singapore

**Keywords:** secondary metabolites, anti-cancer, alternative medicine, polyvinylpolypyrrolidone (PVPP), phenol quenching, cell lines, metabolic profiling, multiple solvents

## Abstract

*Rubia cordifolia* L. (Rubiaceae) is an important plant in Indian and Chinese medical systems. Extracts prepared from the root, stem and leaf have been used traditionally for the management of various diseases. Some of the known effects are anti-inflammation, neuroprotection, anti-proliferation, immunomodulation and anti-tumor. A comparative account of the extracts derived from different organs that lead to the identification of the most suitable solvent is lacking. We explored the presence of phytochemicals, antioxidant activity and anti-proliferative properties of a variety of solvent-based extracts of root, and methanol extracts of stem and leaf of *R. cordifolia* L. The antioxidant potential was determined by DPPH, hydrogen peroxide, nitric oxide and total antioxidant assays. The anti-proliferative nature was evaluated by MTT assay on HeLa, ME-180 and HepG2 cells. The composition of the extracts was determined by UPLC-UV-MS. We found that the root extracts had the presence of higher amounts of antioxidants over the stem and leaf extracts. The root extracts prepared in methanol exhibited the highest cytotoxicity in HepG2 cells. The main compounds identified through UPLC-UV-MS of the methanol extract give credibility to the previous results. Our comprehensive study corroborates the preference given to the root over the stem and leaf for extract preparation. In conclusion, we identified the methanol extract of the root to be the most suited to have bioactivity with anti-cancer potential.

## 1. Introduction

Various chemotherapeutic drugs inherently induce side effects due to a lack of non-specificity towards cancer cells. The search for newer molecules has led to a refreshed look at complementary and alternative medicinal practices [1,2]. A plant-derived anti-cancer molecule is expected to provide a solution owing to its natural source. Hence, many plants are being investigated in view of this vital necessity. Extracts from traditional medicinal plants, such as *Rubia cordifolia* L. may be one of the alternatives available to fill this lacuna. 

Geographically, *R cordifolia* L. is a widely distributed member of the Rubiaceae family. It is traditionally referred to as Indian Madder or Manjith or Manjistha in India and Qiancao in China. The inherent red color of the root is used as a food coloring agent and dye for fiber. Its usage as a phytomedicine has been documented in the traditional Indian medicine systems of Ayurveda and Siddha and traditional Chinese medicine. Broadly, the extracts have been used for the treatment of blood-related conditions, such as hematemesis, epistaxis, spotting, traumatic bleeding and amenorrhea [3]. The extract preparation may be water-based (aqueous) and organic-solvent-based (such as methanol, ethanol, chloroform and dichloromethane). The aqueous extract of the aerial parts effectively controls diarrhea and inflammation in male Swiss albino mice [4]. The aqueous extract of the whole plant limits the rotavirus multiplication in MA-104 cells [5]. Methanol extracts prepared from the root have cardioprotective [6] and anti-cancer activities, as determined in the human epidermoid laryngeal carcinoma cell line (HEp-2) [7], anti-human lymphoma cells (U937) and malignant skin melanoma (A375) [8]. The ethanol extract of the root has been evaluated to be anti-thrombotic and pro-angiogenic [9]. Animal studies using chloroform extract of the whole plant did not disclose any significant anti-tumor activity [10]. 

Many compounds have been identified from *R. cordifolia* that may be responsible for such therapeutic actions [9,11]. The compounds present in the *R. cordifolia* are reviewed by Shan et al. [3], and those with a PubChem ID are consolidated in Table 1. 

Quinones, terpenoids, alkaloids and their derivatives form a major class of compounds with considerable bioactivities. These components are responsible for the various anti-oxidation, anti-inflammation and anti-proliferative bioactivities, among others. Mollugin (derivative of anthraquinone) inhibits pro-inflammatory chemocytokine production [12]. Purpurin is another anthraquinone that gives *R. cordifolia* L antioxidant properties [13]. Alizarin, 6-hydroxyrubiadin, purpurin and rubiadin are expected to be key constituents responsible for analgesic and anti-inflammatory properties [14]. The mode of action of the exhaustive list of compounds has not been elucidated completely as many compounds are solvent-specific and are not available in large quantities. 

Quantified research that directs to the therapeutic usage of specific extraction solvents for different plant organs is still lacking. Further, a comparison among the different extracts prepared from different *R. cordifolia* plant organs remains unattempted so far. Within this frame of reference, we have focused our attention on the antioxidant and anti-proliferative activities of various extracts prepared from *R. cordifolia* and have identified methanol as the most suitable solvent [15]. An in vitro analysis on the cancer cell lines confirmed the methanol extract of the root as the most suitable for pertinent pre-clinical studies.

## 2. Materials and Methods

### 2.1. Plant Collection

The stems and leaves of *R. cordifolia* were freshly collected from Torna fort (18°16′33.86″ N 73°37′21.78″ E) and Mahabaleshwar Forest (17°55′51″ N 73°38′52″ E) located in Maharashtra State, India. Air-dried leaves and stems were separated. The dried samples were pulverized into a coarse powder and stored for further use. The plant was authenticated at Botanical Survey of India, Pune, India center with specimen number MGJRC-1 and a voucher specimen is deposited at the BSI herbarium. 

### 2.2. Preparation of Extracts

All solvents, reagents and standards used were of analytical grade (HiMedia, Mumbai, India). Extracts of powders were prepared in methanol, ethanol or distilled water as described previously [16]. In brief, powders of different plant parts of *R. cordifolia* were extracted with solvent individually by conventional Soxhlet apparatus (Goel Scientific, Vadodara, India) extraction procedure. After the exhaustive extraction, each extract was evaporated to dryness by rotary evaporator (Aditya Scientific, Hyderabad, India). We quenched the polyphenols using polyvinylpolypyrrolidone (PVPP) to determine if antioxidant activity is exclusive to the polyphenols present in the extract. To remove polyphenols from the extracts, they were treated with 10% (*w/v*) PVPP made in respective solvents and kept on a shaking incubator (238019, Thermo Fisher, Waltham, MA, USA) at 37 °C overnight. The polyphenols bind with PVPP and settle at the bottom, while the supernatant contains the polyphenol-free extract [17].

### 2.3. Qualitative Phytochemical Screening of R. cordifolia Constituents 

The preliminary screening of different classes of natural plant constituents was performed. The presence of secondary metabolites viz. alkaloids, saponins, tannins, phenols, glycosides, terpenes, carotenoids and quinones was detected using the standard tests as described below [16,18]. 

#### 2.3.1. Alkaloid Detection

Mayer’s test for alkaloids was performed by treating equivalent volumes of extract with Mayer’s reagent (in-house prepared by dissolving 1.36 g of mercuric chloride (GRM1067, HiMedia, Mumbai, India) and 5 g of potassium iodide (GRM252, HiMedia, Mumbai, India) in 100 mL distilled water), and the subsequent development of cream-colored precipitate implied existence of alkaloid. Dragenforff’s reagent was prepared by dissolving 8 g of bismuth nitrate (RM1221, HiMedia, Mumbai, India) in 20 mL of concentrated nitric acid (GRM6105, HiMedia, Mumbai, India) and 27.2 g of potassium iodide (KI) in 50 mL of distilled water. Both the solutions were kept standing till KIO_3_ crystallized out. The supernatant was decanted, and final volume was adjusted to 100 mL with distilled water. Dragendorff’s test for alkaloids was accomplished by treating equivalent volumes of extract with Dragendorff’s reagent. Subsequent generation of red-colored precipitate suggested presence of alkaloid. Wagner’s reagent was prepared by dissolving 2 g of iodine (GRM1064, HiMedia, Mumbai, India) and 6 g of potassium iodide in 100 mL of distilled water. Wagner’s test for alkaloids was performed by treating equivalent volumes of extract with Wagner’s reagent. Subsequent development of reddish-brown-colored precipitate indicated existence of alkaloid. Hager’s reagent was prepared by dissolving 1 g of picric acid (S026, HiMedia Mumbai, India) in 100 mL of distilled water. Hager’s test for alkaloids was performed by treating equivalent volumes of extract with Hager’s reagent. Subsequent development of yellow-colored precipitate suggested presence of alkaloid. 

#### 2.3.2. Saponin Detection

Saponin was detected by dissolving equivalent quantity of extract in water followed by vigorous shaking. Formation of honeycomb-shaped persistent froth indicated the existence of saponins in the sample. 

#### 2.3.3. Tannin Detection

Tannins were determined by mixing extract with 0.5% aqueous ferric chloride (GRM165-500G, HiMedia, Mumbai, India), and dark green/bluish-green coloration of the sample indicated presence of tannins. 

#### 2.3.4. Phenol Detection

Phenols were determined by adding equivalent volumes of extract to Folin–Ciocalteu reagent (RM10822, HiMedia, Mumbai, India), and blue coloration of sample indicated presence of phenols. 

#### 2.3.5. Glycoside Detection

Glycosides were identified by treating equivalent volumes of extract with glacial acetic acid (AS001, HiMedia, Mumbai, India) and some drops of 5% aqueous ferric chloride (FeCl_3_) and concentrated sulphuric acid (H_2_SO_4_) (AS016-500ML, HiMedia, Mumbai, India). This is known as Keller-Kiliani test. Reddish-brown coloration at the confluence and bluish-green color in top layer solution indicated presence of glycosides. 

#### 2.3.6. Flavonoids Detection

Flavonoids were detected by Shinoda test when to 1 ml of extract, few Mg turnings were added followed by a few drops of concentrated hydrochloric acid (HCl). Development of reddish pink coloration indicated presence of flavonoids.

#### 2.3.7. Terpene Detection

Terpenes were detected by mixing equivalent volumes of extract with chloroform and concentrated sulphuric acid. Reddish-brown coloration at the junction of two solutions suggested the occurrence of terpenes. 

#### 2.3.8. Steroid Detection

Steroids were detected by formation of orange color in solution consisting of equivalent volumes of extract with chloroform, glacial acetic acid and concentrated sulphuric acid. 

#### 2.3.9. Quinone Detection

Presence of quinone was determined by formation of green color upon addition of concentrated hydrogen chloride (RM5955-500ML, HiMedia, Mumbai, India) to the extract [19]. 

#### 2.3.10. Carotenoids Detection

Carotenoids were detected by formation of deep blue color in solution consisting of equivalent volumes of extract with concentrated sulphuric acid (H_2_SO_4_) and a few crystals of iodine. 

### 2.4. Quantification of Phenols

Phenolic content was determined according to the method reported earlier [17]. Briefly, 1 mL of 1 mg/mL extract and gallic acid with the concentrations of 20, 40, 60, 80 and 100 µg/mL was mixed with 0.5 mL of 1N Folin–Ciocalteu reagent and incubated for 5 min, followed by addition of 1 mL of 20% sodium carbonate. After 10 min incubation at room temperature, absorbance was measured at 730 nm. Gallic acid was used as the standard and the phenolic content was expressed as gallic acid equivalent (GAE). The equation of the curve: *y = mx + c* with R^2^ > 0.99. The limit of detection (LOD) and limit of quantification (LOQ) were based on the standard deviation of the blank and calculated using following equations:

(1) LOD=3.3×σ/S 

(2) LOQ=10×σ/S 

where σ is the standard deviation of y-intercepts of the regression line, and S is the slope of the calibration curve. 

### 2.5. Quantification of Flavonoids

Flavonoid content in the extract was determined in accordance with the reported method [20]. In brief, 1 mL of extract and quercetin with the concentration of 100, 200, 300, 400 and 500 µg/mL was mixed with 1.25 mL of distilled water and 75 µL of 5% of sodium nitrite solution incubated for 5 min; subsequently, 150 µL of 10% aluminum chloride (Sigma-Aldrich, Burlington, MA, USA) solution was added. After incubation for 6 min, 500 µL of 1 M sodium hydroxide and 275 µL of distilled water were added to prepare the mixture. The absorbance was recorded at 510 nm. Quercetin was used as the standard, and the flavonoid content is expressed as quercetin equivalent (QE). The equation of the curve: *y = mx + c* with R^2^ > 0.99. The LOD and LOQ were based on the standard deviation of the blank and calculated as described by equations 1 and 2, respectively.

### 2.6. Antioxidant Assays

#### 2.6.1. DPPH Free Radical Scavenging Assay

DPPH (2,2-diphenyl-1-picrylhydrazyl) scavenging activity was measured with spectrophotometric method as described previously [21]. To 0.5 mL extract solution made in respective solvents of concentration ranging from 20 to 100 µg/mL, 1 mL of 0.2 mM DPPH (RM2798, HiMedia, Mumbai, India) made in methanol was added and volume was made up to 2 mL with methanol and incubated for 30 min at room temperature. The absorbance was measured at 517 nm against blank. Ascorbic acid was used as the standard control. The antioxidant activity was presented as IC_50_ value (µg/mL) based on percentage of inhibition of DPPH as calculated in accordance with Equation (3).

(3) $$Percent\;scavenging\;activity=\frac{\left(\left(Acontrol-Asample\right)\times 100\right)}{Acontrol}$$

#### 2.6.2. Hydrogen Peroxide Scavenging Assay

The scavenging effect of hydrogen peroxide was determined as described earlier [22]. Briefly, 1 mL of extract solution of concentration ranging from 20 to 100 µg/mL was treated with 0.6 mL, 40 mM of hydrogen peroxide (Fisher Scientific, Pittsburgh, PA, USA) prepared in phosphate buffer (pH 7.4) for 10 min. The absorbance was read at 230 nm against blank of hydrogen peroxide. Ascorbic acid was used as standard, and the antioxidant activity was presented as IC_50_ value (µg/mL) based on percentage of inhibition of hydrogen peroxide (Equation (3)).

#### 2.6.3. Scavenging Activity of Nitric Oxide

Nitric oxide was generated from sodium nitroprusside, and its scavenging effect was determined as described previously [16]. Briefly, different concentrations ranging from 20 to 100 µg/mL of 1 mL of extract solution and 1 mL (pH 7.4) phosphate buffer were used to prepare 0.5 mL of 10 mM sodium nitroprusside. After incubation for 5 h at 25 °C, 0.5 mL of supernatant liquid was removed and 0.5 mL of Griess reagent (G7921, Thermo Fisher, Waltham, MA, USA) (1 mM) prepared in distilled water was added. The absorbance of the chromophore formed during diazotization of nitric oxide with sulphanilamide and its subsequent coupling with *N*-(1-naphthyl) ethylene–diamine was determined at 546 nm. Ascorbic acid was used as standard, and the antioxidant activity was presented as IC_50_ value (µg/mL) based on percentage of inhibition of nitric oxide (Equation (3)).

#### 2.6.4. Determination of Total Antioxidant Capacity 

The total antioxidant capacity was determined by phosphomolybdate assay [23]. In brief, 1 mL of extract of concentrations ranging from 20 to 100 μg/mL prepared in respective solvents was taken and mixed with 1 mL of reagent containing 0.6 M sulphuric acid, 28 mM sodium phosphate (MB047-250G, Thermo Fisher, Waltham, MA, USA) and 4 mM ammonium molybdate (A7302-100G, Sigma-Aldrich, Burlington, MA, USA). The solution formed was incubated at 95 °C for 90 min, cooled to room temperature and absorbance was noted at 695 nm. Ascorbic acid was used as the standard, and the total antioxidant capacity was calculated as percentage scavenging activity (refer Equation (3)). 

### 2.7. Principal Component Analysis 

Principal component analysis (PCA) was performed to point out the clustering of data into two separated groups, namely PVPP untreated (−PVPP) and treated (+PVPP) extracts. The PCA is a procedure aiming at reducing the dimensionality of the data and allowing the visualization of a large number of variables in a two-dimensional plot [24]. The input data were obtained from quantification of phenol and flavonoid and antioxidant activity (phenol content expressed as mg GAE/g of plant extract; flavonoid content expressed as mg QE/g of plant extract; antioxidant potential by DPPH free radical scavenging expressed as IC_50_; antioxidant potential by hydrogen peroxide scavenging expressed as IC_50_; antioxidant potential by nitric oxide scavenging assay expressed as IC_50_ and total antioxidant capacity expressed as IC_50_) in root-methanol, root-ethanol, root-aqueous, leaf-methanol and stem-methanol extracts. A diagram of the values obtained from each treatment condition was plotted in the bidimensional space, defined by the 1st and 2nd principal component functions (PC1 and PC2, respectively).

### 2.8. Cell Culture and Cytotoxicity

Authenticated cell lines ME-180, HeLa and HepG2 were procured from National Centre for Cell Science, Pune, India. The cells were grown in Roswell Park Memorial Institute-1640, Eagle’s Minimal Essential Medium and Dulbecco’s Modified Eagle Medium media, respectively, and 10% FBS (16000044, Thermo Fisher, Waltham, MA, USA) and 1% antibiotic solution were used for supplementation. Cells were grown in T-25 flasks and were passaged upon confluence using trypsin-EDTA [16]. Nearly 5000 cells were seeded per well in 96-well plate and incubated at 37 °C in 5% CO_2_ incubator and left overnight to enable surface attachment. Cells were treated with extracts (methanol, ethanol and aqueous) with concentrations of 50, 25, 10, 5, 1, 0.5, 0.1, 0.05 mg/mL and left overnight in incubator. 5 mg/mL of MTT per well was added and incubated for 2 h at 37 °C. Formazan crystals were solubilized with 100 μL DMSO and incubated for 10 min. The absorbance was measured at 570 nm and reference at 630 nm.

### 2.9. UPLC-UV-MS Analysis

UPLC-UV-MS phytochemical profiling of root methanol extract (1 mg/ml) was performed on an Agilent 6540 UHD Accurate Mass QTOF MS system (Agilent Technologies, Santa Clara, CA, USA). The separation was performed using a Zorbax 2.1 × 50 mm 1.8 μm column. The gradient applied was: 0.1% formic acid in water (A), acetonitrile (B); 0 min 95% B; 5 min 95% B; 6 min 5% B; 8 min 5% B. Injection volume was 10 μL; flow-rate was 0.2 mL/min. ESI-Q-TOF-MS analysis was performed in the positive and negative ionization modes using the following parameters: mass range 70–1600 *m*/*z*; gas temperature 270 °C; nitrogen flow 11 L/min; nebulizer pressure 45 psig; skimmer 45 V; capillary voltage 4000 V; fragmentor 150 V, fixed collision energy 40 V. Data were processed with Agilent MassHunter 6200 series TOF/6500 series Q-TOF B.09.00 (B9044.0) (Agilent Technologies, Santa Clara, CA, USA).

### 2.10. Statistical Analysis

All experiments were performed in triplicate and the values were expressed as mean ± standard error of mean (SEM). The data were analyzed by Student–Newman–Keuls test using Sigma Plot version 14 (Systat Software Inc., Palo Alto, CA, USA), and IC_50_ values were calculated using OriginPro, version 2021 (OriginLab Corporation, Northampton, MA, USA).

## 3. Results

### 3.1. Qualitative Analysis of Secondary Metabolites of R. cordifolia Extracts

The methanol extract of *R*. *cordifolia* root had alkaloids, tannins, phenols, flavonoids and terpenes (Table 2). In comparison, while the ethanol extract lacked tannins, the aqueous extract had saponins and glycosides. Considering the maximally reported usage of methanol extracts for roots, we evaluated methanol extracts of leaves and stems in the same way. In contrast to the methanol extracts of roots, the methanol extracts of leaves had glycosides and quinones, while the stem-methanol extracts had quinones and carotenoids.

### 3.2. Quantification of Phenols and Flavonoids in Extracts

Standard calibration curves were plotted for the quantification of phenols in extracts. The plot for standard gallic acid for both PVPP-untreated and -treated was linear, with correlation coefficients (R^2^) equal to 0.9916 and 0.99, respectively. The regression equations for PVPP-untreated and -treated were y = 0.0093x + 0.0436 and y = 0.0062x + 0.0335, respectively, with an LOD under 10 mg/g and LOQ under 30 mg/g for both. Similarly, standard quercetin calibration plots were obtained as linear with R^2^ of 0.9986 and 0.991, and regression equations of y = 0.0014x + 0.0067 and y = 0.0012x + 0.0308 for PVPP-untreated and -treated, respectively. The LODs were under 20 mg/g and LOQs were under 40 mg/g for both.

Significant levels of difference were observed in all the root extracts post-PVPP treatment for the phenols and flavonoids. The ethanol and methanol extracts of roots had the highest phenol and flavonoid content, respectively, compared to the other extracts for 1 mg/mL concentrations of extracts (Table 3). The roots had the highest phenol and flavonoid content among the methanol extracts of different organs of *R. cordifolia* L. 

### 3.3. Root Extracts Have Better Antioxidant Activity Than Leaf and Stem Extracts

**Figure 1:**
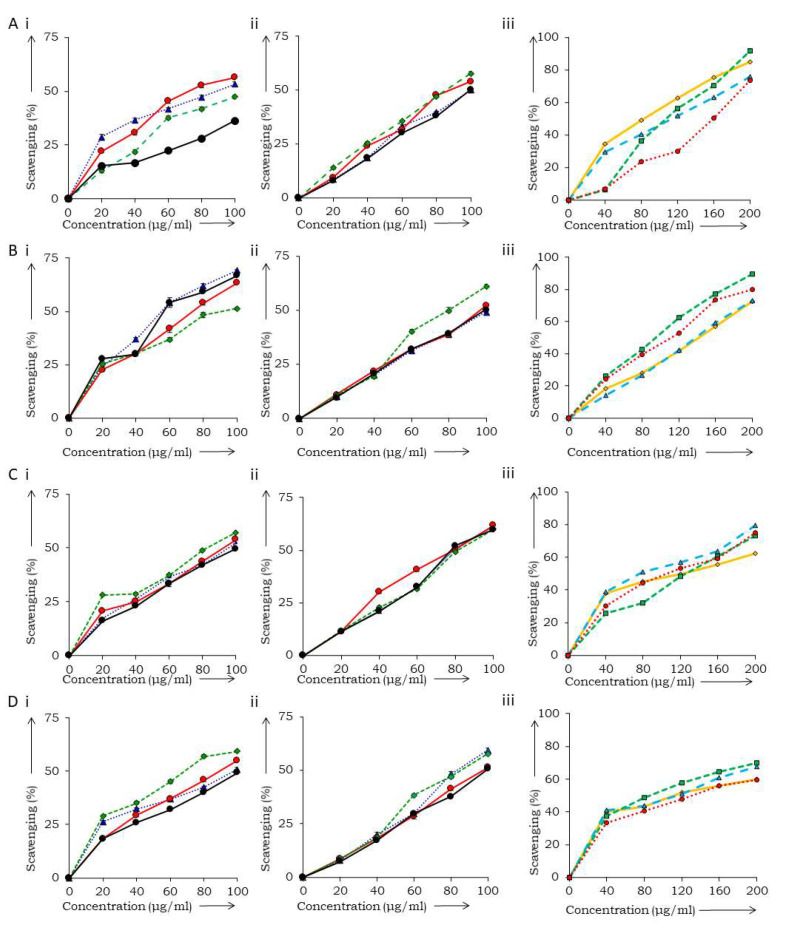
In vitro antioxidant assays (**A**) DPPH assay, (**B**) hydrogen peroxide scavenging assay, (**C**) nitric oxide scavenging assay, (**D**) total antioxidant assay of *R. cordifolia* root without (i) and with (ii) polyvinylpolypyrrolidone (PVPP), where: blue—ethanol extract, red—methanol extract, dark green—aqueous extract, black—ascorbic acid and (iii) leaf and stem, where: green—leaf-methanol extract (−PVPP), orange—methanol extract (+PVPP), red—stem-methanol extract (−PVPP), blue—stem-methanol extract (+PVPP).

The percentage of scavenging activity of the root-ethanol extract in 2,2-diphenyl-1-picrylhydrazyl (DPPH) and hydrogen peroxide assays is less in absence of the PVPP treatment, while higher IC_50_ values were obtained in the presence of PVPP in nitric oxide and total antioxidant assays (Figure 1 and Table 4). With the post-treatment of root extracts by PVPP, the aqueous extract was found to be 84%, 81% and 84% more potent in DPPH, hydrogen peroxide and total antioxidant assay, respectively. The methanol extracts of leaf and stem showed higher IC_50_ in all the assays. Considering the absence of significant levels of antioxidant activities in the methanol extracts of leaves and stems, we continued with the extracts of root for further assays.

### 3.4. Principal Component Analysis of R. cordifolia Phenol, Flavonoid and Antioxidant Levels in PVPP Untreated and Treated Extracts

The data obtained by the quantification of the phenols and flavonoids with antioxidant levels of *R. cordifolia* among PVPP-untreated and -treated extracts have been used to perform a principal components analysis (PCA) (Table 5).

As shown in Table 6, the first principal component was highly correlated with flavonoid content and antioxidant levels by H_2_O_2_ scavenging activity (Antioxidant_H_2_O_2_) variables, while the second principal component was highly correlated with antioxidant levels by NO scavenging activity (Antioxidant_NO) variable (highest score coefficients in absolute value).

**Figure 2:**
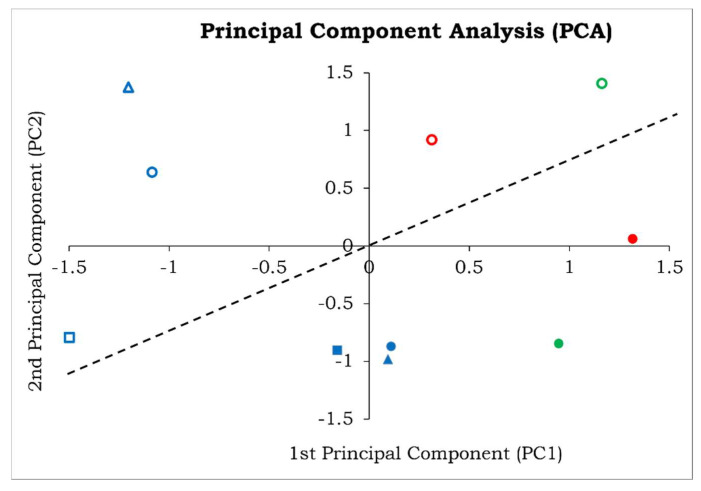
Principal components analysis (PCA) of *R. cordifolia* L antioxidant activity in PVPP untreated (–PVPP, empty symbols) and treated (+PVPP, solid plain symbols) extracts. The different samples and extractions conditions have been indicated with different shape and color symbols: methanol = circle; ethanol = triangle; aqueous = square; root = blue; leaf = red and stem = green.

The first and the second principal components explain together 77.44% of the total observed variance, which is a considerable value. The PCA showed a clear separation between the -PVPP and +PVPP data (Figure 2), as better evidenced by the dotted line added in the plot.

Negative PC1 values correlated to the flavonoid content and Antioxidant_H_2_O_2_ variables were mostly associated with the root samples (blue symbols). On the other hand, PVPP-untreated (empty symbols) and -treated (solid plain symbols) roots were markedly separated by the dotted line, indicating that the flavonoid content, H_2_O_2_ and NO antioxidant activities are different. The stem samples (green symbols) have different PC2 values correlated to Antioxidant_NO scavenging activity, and leaf samples (red symbols) have different PC1 and PC2 values since they are separated by the dotted line.

The methanol extracts (circle symbols) have mostly positive PC1 values but different PC2 values, indicating a difference in the Antioxidant_NO activity related to the PVPP treatment. The aqueous extracts (square symbols) have negative PC1 and similar PC2 values, indicating a similar Antioxidant_NO activity independent from the PVPP treatment. The ethanol extracts (triangle symbols) have both PC1 and PC2 values, indicating different flavonoid content, Antioxidant_H_2_O_2_ and NO activities related to the PVPP treatment.

### 3.5. Plant Extracts Are Cytotoxic to Cancer Cells

**Figure 3:**
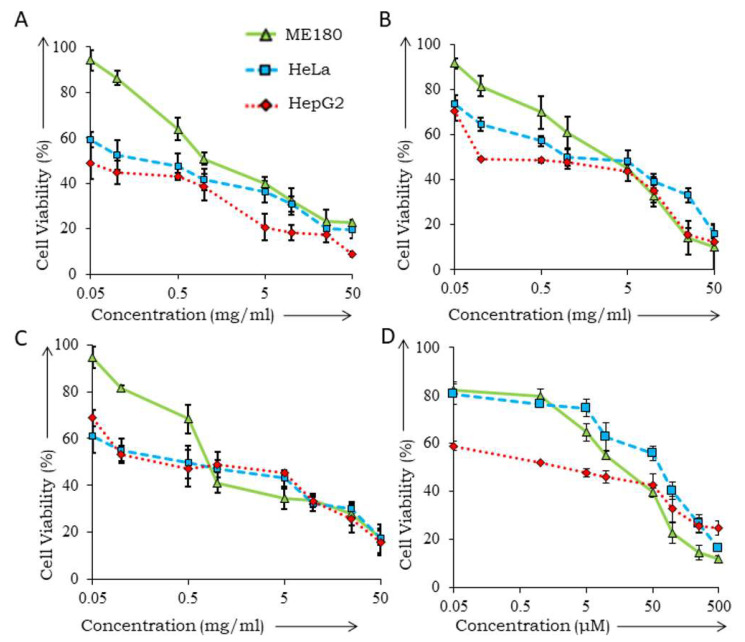
Comparative cell viability assay on three cell lines: HeLa (blue intermittent line with square marker), ME-180 (green continuous line with triangle marker) and HepG2 (red dotted line with diamond marker) using *R. cordifolia* extracts (**A**) methanol extract, (**B**) ethanol extract, (**C**) aqueous extract and (**D**) 5-Flurouracil. The cell viability is relative to the vehicle control (cells treated with solvent in equivalent amounts of respective extract). Results were expressed as the mean *±* SD of three independent experiments.

**Figure 4:**
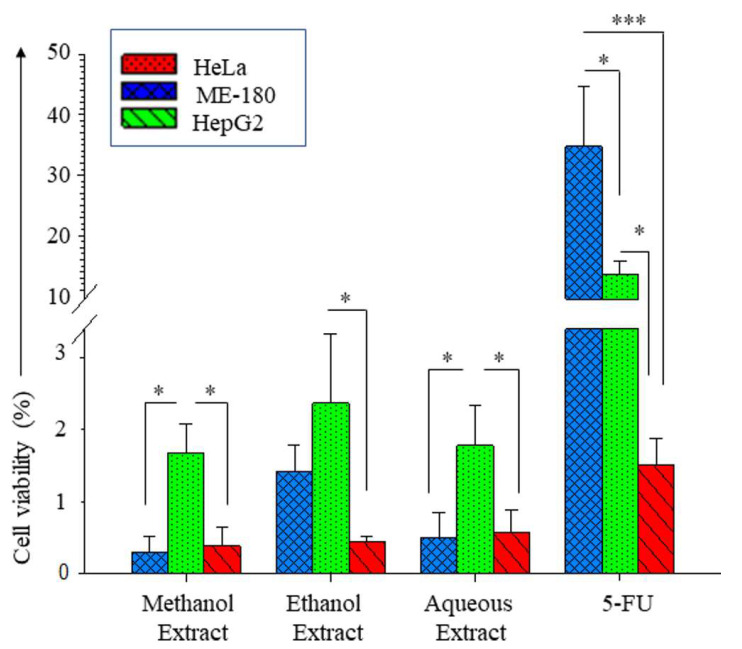
Comparison among the IC_50_ of HeLa (blue), ME-180 (green) and HepG2 (red) cells upon treatment with *R. cordifolia* root extracts of methanol, ethanol, aqueous extract and 5-Flurouracil (5-FU). Results were expressed as the mean *±* SD of three independent experiments, test of significance by ANOVA, wherein * and *** represent statistical significance of *p* < 0.05 and *p* < 0.001, respectively.

Cancer cell lines ME-180, HeLa and HepG2 were exposed to various concentrations of extracts and standard drug (5-Flurouracil) to determine the cell viability by MTT cell proliferation assay. HeLa and HepG2 cells were susceptible to any of the extracts at similar concentrations (Figure 3 and Figure 4). The root-methanol extract was more potent than other extracts for HeLa (IC_50_ of 0.29 ± 0.23 mg/mL) and HepG2 (IC_50_ of 0.39 ± 0.26 mg/mL) (Table 7). 5-Flurouracil (5-FU) was most toxic to HepG2 cells (IC_50_ of 1.51 ± 0.38μM), and the levels of toxicity were significantly lower than those in the other cell lines evaluated.

### 3.6. UPLC-UV-MS Phytochemical Profiling of Methanol Extract of R. cordifolia

**Figure 5:**
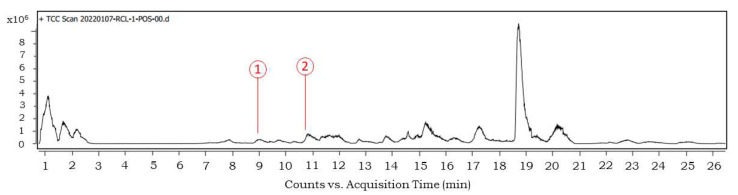
UPLC-UV-MS analysis on positive node for methanol extract with identified peaks of 1. Pseudopurpurin, 2. Morindaparvin A.

**Figure 6:**
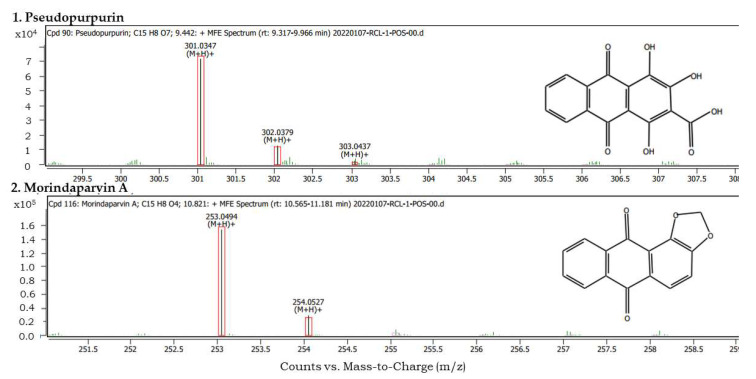
Chromatograms and structure (inset) of compounds identified by UPLC-UV-MS.

To identify the compounds responsible for anti-proliferative potential, the composition of the root methanol extract was evaluated by UPLC-UV-MS analysis. A number of secondary metabolites were detected (Supplementary Table S1). Out of them, the structures of two of the signature compounds from *R. cordifolia* L. were used to compare with the existing PubChem database (Figure 5 and Figure 6). In addition, the formula, score, mass and CAS numbers with retention time are listed in Table 8. 

## 4. Discussion

The utility of secondary metabolites for human health has achieved high recognition owing to their promising usage in traditional knowledge-based medication for centuries. *R. cordifolia* L produces a range of secondary metabolites that have been evaluated for various illnesses. In the present study, we have evaluated three solvent systems for roots and methanol as a solvent for stems and leaves to extract secondary metabolites from *R. cordifolia* L. The suitability of methanol extracts in antioxidant assays prompted us to evaluate methanol extracts of stems and leaves for phytochemical analysis. Nevertheless, the antioxidant levels of roots were noticed to be higher than stems and leaves.

Phenols are a major antioxidant group present in plants. We detected significant amounts of phenols in the ethanol extract of root, followed by the methanol extract of leaf. Flavonoids are the largest group of natural phenolics that possess tremendous free radical scavenging properties and, hence, antioxidant potential. Our method of Soxhlet extraction led to an increased release of phenols and flavonoids.

The presence of antioxidants in the extract is crucial for usage as an anti-proliferative agent. The results of the DPPH assay for the ethanol extract of root reported by Zhang et al. [25] were in the range of 23.88 to 65.23 µg/mL. They used an ultrasonic-assisted extraction process. These values are much lower than the presently reported values in the range of 88.5 to 98.26 µg/mL. We believe the suitability of the extraction method and the mother plant selection are the drivers of differential results. Basu and Hazra [26] reported a range of 153.7 to 310.3 µg/mL for methanol and aqueous extracts of root as evaluated by a nitric oxide assay. They used the filtrate of the directly solubilized extracts in the respective solvents. Our results have a different range, possibly due to our choice of method of the Soxhlet exhaustive extraction process.

The antioxidant activity of the plant extract is attributed to various secondary metabolites, including polyphenols. Studies pertaining to the significance of polyphenols have emphasized their influence on the antioxidant results [27,28,29]. We propose to present the case that the antioxidant activity observed in *R. cordifolia* L. is not entirely due to polyphenols. To prove that the determined antioxidant activity is not exclusive to the polyphenols present in the extract and is contributed to by other secondary metabolites as well, we quenched the polyphenols using PVPP. Rantunge et al., 2017 have demonstrated the quenching effect of PVPP on different polyphenols, and it clearly shows remarkable differences [17]. The precipitation allows the removal of any complex of PVPP-polyphenols. A comparison of the PVPP-untreated and -treated extracts by the same antioxidant assays proved that there are other compounds responsible for antioxidant properties as well. We are reporting for the first time the results pertaining to *R*. *cordifolia* root extracts (ethanol, methanol and aqueous) treated with PVPP for antioxidant assays. Even after the removal of phenols and flavonoids, the antioxidant activity of the extract is not hampered. This suggests the involvement of other non-phenolic secondary metabolites in bringing about the antioxidant potential. The PCA correlated the phenol, flavonoid and antioxidant levels, as evaluated by hydrogen peroxide and nitric oxide scavenging assays. Our evaluation of *R. cordifolia* leaves and stems demonstrates that the root is more suited to be used for antioxidant properties. The high prevalence of antioxidant compounds in root extracts may be utilized for the anti-proliferative process in certain cancers [30,31]. The anti-proliferative assay corroborated the suitability of the methanol extract of the root for anti-cancer activity. The sensitivity of HepG2 towards 5-Flurouracil as compared to other cell lines was not reflected for plant extracts as similar toxicity was observed in the ME180 and HeLa for cell lines, suggesting its usage for the management of multiple cancers. The cytotoxicity may be mediated by reactive oxygen species, as indicated in laryngeal squamous cell carcinoma HEp-2 cells [7]. However, the apoptotic pathway responsible for cell toxicity needs further elucidation. The isolation of suitable cancer-specific bioactive compounds is necessary, or else it may yield a negative result [10].

Our results of UPLC-UV-MS identified some previously reported compounds and some new compounds from Rubia plants. Pseudopurpurin (anthraquinone) is a characteristic natural red-color compound present in the roots of *R. cordifolia* and Rubiaceae family members. It is a derivative of purpurin (pseudopurpurin is purpurin 3-carboxylic acid). It improves bone geometry [32] and selectively exhibits tumor inhibitory potential [33]. Morindaparvin A is reported to be an antileukemic anthraquinone and is chemically derived from alizarin (1,2-methylenedioxyanthraquinone by synthesis from alizarin) [34]. We report its presence in *R. cordifolia* for the first time. It is possible that its presence was not detected previously or was not considered as it is a derivative of alizarin. These preliminary findings require a detailed supplemental study for verification before confirmation.

The presence of multiple compounds in the methanol extract that are established to be cytotoxic to cancer cells supports our results. However, the validation of cytotoxic activities requires independent assays.

## 5. Conclusions

*R. cordifolia* L. is a widely used plant for its significant medicinal value. This is attributed to the presence of unique secondary metabolites in *R. cordifolia* L. Exhaustive methods of extraction lead to an increase in the retrieval of secondary metabolites, as observed in our research endeavor. This work provides the initial steps required in selecting the suitable solvents for *R. cordifolia* extract preparations. Our study has revealed the presence of a high quantity of antioxidants in the root, stem and leaf extracts of *R*. *cordifolia*. The antioxidant levels in the root, stem and leaf provide a comparative benchmark for further exploration. The results obtained for the antiproliferative assay make the extracts valuable to medicinal practitioners. Identification of different compounds may help in determining a metabolite signature characteristic of *R. cordifolia*. The individual compounds need to be evaluated to verify the extent of the utility of the antioxidant nature for identifying a potential anti-cancer agent. In summary, the medicinal value imparted by the extracts is comprehensively documented for its usage in anti-cancer research.

## Figures and Tables

**Table 1:** Selected compounds identified from *R. cordifolia* L.

No.	Chemical Compounds	PubChem ID	Molecular Formula	Molecular Weight (g/mol)	Isolated from
1	6-methoxygeniposidic acid	50998059	C_17_H_24_O_11_	404.4	Root
2	Rubiprasin A	21594201	C_32_H_52_O_5_	516.799	Root
3	Rubiprasin B	21594133	C_32_H_52_O_4_	500.8	Root
4	Rubiarbonol A	12019473	C_30_H_50_O_4_	474.7	Root
5	Rubiarbonol B	12019474	C_30_H_50_O_3_	458.7	Root
6	Rubiarbonol C	21672545	C_32_H_52_O_5_	516.799	Root
7	Rubiarbonol D	21672546	C_32_H_52_O_5_	516.799	Root
8	Rubiarbonol E	21582934	C_30_H_50_O_4_	474.7	Root
9	Rubiarbonol F	21582935	C_30_H_50_O_5_	490.7	Root
10	1,8-dihydroxyanthraquinone	2950	C_14_H_8_O_4_	240.21	Root
11	1-hydroxy 2-methoxy anthraquinone	80103	C_15_H_10_O_4_	254.24	Root
12	1,3- dimethoxy 2- carboxy anthraquinone	129670266	C_17_H_12_O_6_	312.27	Root
13	1, 5-dihydroxy 2- methylanthraquinone	182449	C_15_H_10_O_4_	254.24	Root
14	Pseudopurpurin	442765	C_15_H_8_O_7_	300.22	Root
15	Dihydromollugin	10779560	C_17_H_18_O_4_	286.32	Root
16	Munjistin	160476	C_15_H_8_O_6_	284.22	
17	1-hydroxy-2-hydroxymethyl-9,10-anthraquinone	32209	C_15_H_10_O_4_	254.24	Root
18	Mollugin	124219	C_17_H_16_O_4_	284.31	Root
19	2-methyl-1,3,6-trihydroxy-9,10-anthraquinone	5319801	C_15_H_10_O_5_	270.24	Root
20	Rubioncolin B	14777446	C_31_H_24_O_10_	556.5	Root
21	Rubilactone	132415	C_15_H_10_O_5_	270.24	Root
22	1- hydroxy-2 carboxy 3-methoxyanthraquinone	129670276	C_16_H_10_O_6_	298.25	Root
23	Oleanolic acid acetate	6708573	C_32_H_50_O_4_	498.7	Root
24	Hederagenin	73299	C_30_H_48_O_4_	472.7	
25	Β-sitosterol	222284	C_29_H_50_O	414.7	Root
26	Rubiasin A	101064500	C_15_H_16_O_2_	228.29	Root, Stem Root, Stem
27	Rubiasin B	101064501	C_15_H_16_O_2_	228.29	
28	Rubiasin C	101064502	C_15_H_16_O_2_	228.29	
29	1-hydroxy-2-methylanthraquinone	160817	C_15_H_10_O_3_	238.24	Root
30	1,4-dihydroxy-2-methylanthraquinone	99300	C_15_H_10_O_4_	254.24	Root
31	2-methylanthraquinone	6773	C_15_H_10_O_2_	222.24	Root
32	Alizarin	6293	C_14_H_8_O_4_	240.21	Root
33	Rubiadin	124062	C_15_H_10_O_4_	254.24	Root
34	Purpurin	6683	C_14_H_8_O_5_	256.209	Root
35	1,4-dihydroxy-2-methyl-5-methoxyanthraquinone	12714658	C_16_H_12_O_5_	284.26	Root
36	Ruberythric acid	92101	C_25_H_26_O_13_	534.5	Root
37	Lucidine primeveroside	160180	C_26_H_28_O_14_	564.5	Root
38	2,3-dihydroxyanthraquinone	11391150	C_15_H_10_O_4_	254.24	Root
39	1,3-dimethoxyanthraquinone	361511	C_16_H_12_O_4_	268.26	Root
40	3-methoxymollugin	46187191	C_18_H_18_O_5_	314.3	Root
41	Xanthopurpurin	196978	C_14_H_8_O_4_	240.21	Root
42	Methyl 1,4-bisglucosyloxy-3-prenyl-2-naphthoate	10031663	C_29_H_38_O_14_	610.6	Root
43	Physcion	10639	C_16_H_12_O_5_	284.26	Root
44	Nordamnacanthal	160712	C_15_H_8_O_5_	268.22	Root
45	Quinizarin (1,4-dihydroxy-6-methyl-anthraquinone)	6688	C_14_H_8_O_4_	240.21	Root
46	1,4-dihydroxy-2- naphthoic acid	671	C_11_H_8_O_4_	204.18	Root
47	Furomollugin	10354359	C_14_H_10_O_4_	242.23	Root
48	2-methyl-1, 3, 6-trihydroxy-9, 10-anthraquinone	5319801	C_15_H_10_O_5_	270.24	Root
49	RA-I	14390137	C_40_H_48_N_6_O_10_	772.8	Root
50	[Gly-1]ra-vii	10440096	C_40_H_48_N_6_O_9_	756.8	Root
51	[Gly-2]ra-vii	12098468	C_40_H_48_N_6_O_9_	756.8	Root
52	RA-III	14390141	C_41_H_50_N_6_O_10_	786.9	Root
53	RA-V	13361282	C_40_H_48_N_6_O_9_	756.8	Root
54	RA-XXIV	24881308	C_42_H_51_N_7_O_10_	813.9	Root
55	RA-VIII	152772187	C_41_H_50_N_6_O_10_	786.9	Root
56	RA-X	6444175	C_43_H_52_N_6_O_11_	828.9	Root
57	RA-XI	131676023	C_42_H_50_N_6_O_11_	814.9	Root
58	RA-XII	10373581	C_46_H_58_N_6_O_14_	919	Root
59	RA-XIII	14999350	C_48_H_60_N_6_O_16_	977	Root
60	RA-XVI	5320896	C_47_H_58_N_6_O_16_	963	Root
61	RA-XVII	102355358	C_41_H_50_N_6_O_9_	770.9	Root
62	RA-XVIII	25033039	C_41_H_50_N_6_O_10_	786.9	Root
63	RA-XIX	24829365	C_44_H_56_N_6_O_9_	812.9	Root
64	RA-XX	24829366	C_42_H_52_N_6_O_9_	784.9	Root
65	RA-XXI	24861920	C_41_H_50_N_6_O_9_	770.9	Root
66	RA-XXII	24862183	C_41_H_50_N_6_O_10_	786.9	Root
67	Rubicoumaric acid	5377693	C_39_H_54_O_6_	618.8	Whole Plant
68	Rubifolic acid	91895456	C_30_H_48_O_4_	472.7	Whole Plant
69	1-hydroxy-9,10-anthraquinone	8512	C_14_H_8_O_3_	224.21	Root
70	2-carbamoyl-3-methoxy-1,4- naphthoquinone	91825839	C_11_H_7_NO_4_	217.18	Root
71	N-nonadecane	12401	C_19_H_40_	268.5	Root
72	2,6-dihydroxyanthraquinone	6776	C_14_H_8_O_4_	240.21	Root
73	N-heptadecane	12398	C_17_H_36_	240.5	Root
74	Rubiatriol	21582929	C_30_H_50_O_3_	458.7	Root
75	Epoxymollugin	24814354	C_17_H_16_O_5_	300.3	Root
76	1,6-dihydroxy-2-methyl-9,10-anthraquinone	124063	C_15_H_10_O_4_	254.24	Root
77	Citric acid	311	C_6_H_8_O_7_	192.12	
78	Malic acid	525	C_4_H_6_O_5_	134.09	
79	Palmitic acid	985	C_16_H_32_O_2_	256.42	
80	1-hydroxy-2, 7- dimethylanthraquinone	1382	C_16_H_12_O_3_	252.26	
81	Emodin	3220	C_15_H_10_O_5_	270.24	
82	Eugenol	3314	C_10_H_12_O_2_	164.2	
83	Alizarin	6293	C_14_H_8_O_4_	240.21	
84	Quinic acid	6508	C_7_H_12_O_6_	192.17	
85	2-methyl anthraquinone	6773	C_15_H_10_O_2_	222.24	
86	Vanillic acid	8468	C_8_H_8_O_4_	168.15	
87	1-hydroxyanthraquinone	8512	C_14_H_8_O_3_	224.21	
88	Lucidin	10163	C_15_H_10_O_5_	270.24	
89	Naphthohydroquinone	11305	C_10_H_8_O_2_	160.17	
90	Tricosanoic acid	17085	C_23_H_46_O_2_	354.6	
91	Ursolic acid	64945	C_30_H_48_O_3_	456.7	
92	Atraric acid	78435	C_10_H_12_O_4_	196.2	
93	Friedelinol	101341	C_30_H_52_O	428.7	
94	Soranjidiol	124063	C_15_H_10_O_4_	254.24	
95	Lariciresinol	332427	C_20_H_24_O_6_	360.4	
96	Naphthaquinone	377214	C_13_H_11_NO_4_	245.23	
97	Anethole	637563	C_10_H_12_O	148.2	
98	Geraniol	637566	C_10_H_18_O	154.25	
99	Geranyl acetate	1549026	C_12_H_20_O_2_	196.29	
100	Scopoletol	5280460	C_10_H_8_O_4_	192.17	
101	Rosmarinic acid	5281792	C_18_H_16_O_8_	360.3	
102	Daucosterol	5742590	C_35_H_60_O_6_	576.8	
103	1-hydroxy 2-methyl anthraquinone	10250776	C_25_H_26_O_5_	406.5	
104	Rubicordifolin	11786393	C_33_H_28_O_9_	568.6	
105	Oleanolic acid	12313704	C_30_H_46_O_3_	454.7	
106	1, 4-dihydroxy 2- methylanthraquinone	12488527	C_16_H_12_O_5_	284.26	
107	1-Hydroxy-2-(methoxycarbonyl)-3-[(methoxycarbonyl)methyl]-9,10-anthraquinone	13793380	C_19_H_14_O_7_	354.3	
108	Rubiatriol	21582929	C_30_H_50_O_3_	458.7	
109	Rubiprasin B	21594133	C_32_H_52_O_4_	500.8	
110	Rubiprasin A	21594201	C_32_H_52_O_5_	516.8	
111	Rubiarbonol C	21672545	C_32_H_52_O_5_	516.8	
112	1, 4- dihydroxy 2- methyl 5-methoxy anthraquinone	23626543	C_20_H_16_O_7_	368.3	
113	2′-hydroxymollugin	46187192	C_17_H_16_O_5_	300.3	
114	Methyl 6-hydroxy-3-methoxy-2,2-dimethyl-3,4-dihydrobenzo[h]chromene-5-carboxylate	5319476	C_18_H_18_O_5_	316.3	
115	Methyl 3,6-dihydroxy-4-methoxy-2,2-dimethyl-3,4-dihydrobenzo[h]chromene-5-carboxylate	5319446	C_18_H_20_O_6_	332.3	
116	2-methyl-1, 3, 6-trihydroxy-9, 10- anthraquinone	70698136	C_29_H_32_O_15_	620.6	
117	Rubifolic acid	72994727	C_30_H_48_O_4_	472.7	
118	2-Acetoxy-1,5-dihydroxy-7-methylanthraquinone	100994924	C_17_H_12_O_6_	312.27	
119	1, 3- dimethoxy 2-carboxy anthraquinone	129670266	C_17_H_12_O_6_	312.27	
120	Rubicordin A	132553188	C_46_H_60_N_6_O_14_	921	
121	Rubicordin B	132553189	C_47_H_62_N_6_O_14_	935	
122	Rubicordin C	132553190	C_42_H_54_N_6_O_9_	786.9	
123	2, 6-methylanthraquinone	155490709	C_25_H_28_O_6_	424.5	
124	Sitosteryl acetate	348285530	C_29_H_50_O	414.71	
125	Sitostenone	60123241	C_29_H_48_O	412.7	

**Table 2:** Phytochemical screening of root, leaf and stem extracts of *R. cordifolia*.

S.No.	Detection	Assays	Root			Leaf	Stem
			Methanol Extract	Ethanol Extract	Aqueous Extract	Methanol Extract	Methanol Extract
1	Alkaloids	Mayer’s test	−	−	−	+	+
2	Alkaloids	Dragendorff’s test	+	+	+	+	+
3	Alkaloids	Wagner’s test	+	−	−	+	+
4	Alkaloids	Hager’s test	−	−	−	+	+
5	Saponins	Foam test	−	−	+	−	−
6	Tannins	Ferric chloride test	+	−	−	+	−
7	Phenols	Folin–Ciocalteu reagent test	+	+	+	+	+
8	Glycosides	Keller–Kiliani test	−	−	+	+	−
9	Flavonoids	Shinoda test	+	+	+	+	+
10	Terpenes	Chloroform-Sulphuric acid test	+	+	+	+	+
11	Steroids	Liebermann–Burchard test	−	−	−	−	−
12	Quinones	Hydrochloride test	−	−	−	+	+
13	Carotenoids	Iodine crystal test	−	−	−	−	+

+ Present; − absent.

**Table 3:** Quantification of phenol and flavonoid contents in extracts of *R*. *cordifolia*.

Extracts in Solvent	PVPP ‘+’ = presence of PVPP, ‘−‘ = absence of PVPP	Phenol Content (mg GAE/g of Plant Extract)	Flavonoid Content (mg QE/g of Plant Extract)
Root-Methanol	−	43.34 ± 0.27 ^a,b,c^	369.69 ± 1.49 ^a,b,c^
	+	6.59 ± 0.73	55.28 ± 2.7
Root-Ethanol	−	74.31 ± 0.16 ^a,d^	334.9 ± 1.8 ^a,d^
	+	5.46 ± 0.25	49.64 ± 3.11
Root-Aqueous	−	67.14 ± 0.11 ^a^	177.05 ± 3.6 ^a^
	+	6.80 ± 0.25	37.08 ± 1.54
Leaf-Methanol	−	35.12 ± 0.32	55.1 ± 0.46 ^a^
	+	#	#
Stem-Methanol	−	26.87 ± 0.23	49.19 ± 0.61
	+	#	#

Phenol content (gallic acid equivalent, GAE) and flavonoid content (quercetin equivalent, QE) is expressed as mean ± SEM (n = 3); ^a–d^ column-wise values with different superscripts of this type indicate significant difference (*p* < 0.001) ^a^ between −PVPP and +PVPP for same solvent, ^b–d^ for −PVPP for different solvents, ^b^ between methanol and ethanol, ^c^ between methanol and aqueous ^d^ and between ethanol and aqueous. # post-PVPP values were not detectable by spectrophotometer at the concentration tested.

**Table 4:** IC_50_ values of DPPH, hydrogen peroxide, nitric oxide and total antioxidant assay of *R*. *cordifolia*. Results were expressed as the mean *±* SD of three independent experiments. Significant difference between without PVPP and with PVPP representing *p* < 0.001, *p* < 0.01, *p* < 0.05 is by ***, ** and *, respectively. *R. cordifolia* extracts were tested at concentrations of 20, 40, 60, 80, 100 μg/mL.

	DPPH		Hydrogen Peroxide Scavenging Activity		Nitric Oxide Scavenging Activity		Total Antioxidant Capacity	
Extracts	IC_50_ (µg/mL)	IC_50_ (µg/mL)	IC_50_ (µg/mL)	IC_50_ (µg/mL)	IC_50_ (µg/mL)	IC_50_ (µg/mL)	IC_50_ (µg/mL)	IC_50_ (µg/mL)
PVPP	-	+	-	+	-	+	-	+
Root-Methanol	79.1 ± 1.92 **	89.47 ± 0.79	74.5 ± 1.38 ***	97.71 ± 1.69	94.53 ± 1.84 **	78.46 ± 0.7	88.62 ± 1.05 **	97.52 ± 0.88
Root-Ethanol	88.5 ± 2.68 **	98.26 ± 0.73	61.2 ± 2.12 ***	101.14 ± 1.52	95.11 ± 0.74 ***	82.17 ± 0.51	101.15 ± 1.77 **	85.92 ± 0.74
Root-Aqueous	99.97 ± 2.09 **	85.53 ± 1.01	92.97 ± 2.31	80.85 ± 1.89	85.49 ± 0.82	84.23 ± 0.75	71.86 ± 0.3 **	85.14 ± 0.81
Leaf-Methanol	115.76± 0.85 *	84.63 ± 0.03	96.35 ± 1.62 **	146.98 ± 7.13	126.86 ± 1.14	118.99 ± 2.16	91.84 ± 4.24 *	117.95 ± 0.58
Stem-Methanol	153.12± 1.19	112.75 ± 0.09	109.02 ± 1.62	138.41 ± 0.69	111.16 ± 1.36	86.17 ± 0.53	134.83 ± 2.05	103.91 ± 0.78
Ascorbic Acid	159.34 ± 3.41 ***	100.42 ± 1.25	64.49 ± 0.51 *	99.12 ± 2.7	100.01 ± 0.6 *	86.35 ± 0.39	104.26 ± 0.62 *	100.29 ± 1.4

Results were expressed as the mean *±* SD of three independent experiments, test of significance among PVPP untreated and treated extracts by ANOVA, wherein *, ** and *** represent statistical significance of *p* < 0.05, *p* < 0.01 and *p* < 0.001, respectively.

**Table 5:** Consolidated data used for PCA.

Extracts in Solvent	PVPP ‘+’ = presence of PVPP, ‘−‘ = absence of PVPP	Phenol Content	Flavonoid Content	DPPH Free Radical Scavenging Assay	H_2_O_2_ Scavenging Activity	NO Scavenging Activity	Total Antioxidant Capacity
		mg GAE/g of Plant Extract	mg QE/g of Plant Extract	IC_50_ (µg/mL)	IC_50_ (µg/mL)	IC_50_ (µg/mL)	IC_50_ (µg/mL)
Root- Methanol	−	43.34 ± 0.27	369.69 ± 1.49	79.1 ± 1.92	74.5 ± 1.38	94.53 ± 1.84	88.62 ± 1.05
	+	6.59 ± 0.73	55.28 ± 2.7	89.47 ± 0.79	97.71 ± 1.69	78.46 ± 0.7	97.52 ± 0.88
Root- Ethanol	−	74.31 ± 0.16	334.9 ± 1.8	88.49 ± 2.68	61.2 ± 2.12	95.11 ± 0.74	101.15 ± 1.77
	+	5.46 ± 0.25	49.64 ± 3.11	98.26 ± 0.73	101.14 ± 1.52	82.17 ± 0.51	85.92 ± 0.74
Root- Aqueous	−	67.14 ± 0.11	177.05 ± 3.6	99.976 ± 2.01	92.97 ± 2.31	85.49 ± 0.82	71.86 ± 0.3
	+	6.80 ± 0.25	37.08 ± 1.54	85.53 ± 1.01	80.85 ± 1.89	84.23 ± 0.75	85.14 ± 0.81
Leaf- Methanol	−	35.12 ± 0.32	55.1 ± 0.46	115.76 ± 0.85	96.35 ± 1.62	126.86 ± 1.14	91.84 ± 4.24
	+	#	#	84.63 ± 0.03	146.98 ± 7.13	118.99 ± 2.16	117.95 ± 0.58
Stem- Methanol	−	26.87 ± 0.23	49.19 ± 0.61	153.12 ± 1.19	109.02 ± 1.62	111.16 ± 1.36	134.83 ± 2.05
	+	#	#	112.75 ± 0.09	138.41 ± 0.69	86.17 ± 0.53	103.91 ± 0.78
Ascorbic Acid	−	NA	NA	159.34 ± 3.41	64.49 ± 0.51	100.01 ± 0.6	104.26 ± 0.62
	+	NA	NA	100.42 ± 1.25	99.12 ± 2.7	86.35 ± 0.39	100.29 ± 1.4

# post-PVPP values were not detectable by spectrophotometer at the concentration tested. NA—Not Applicable.

**Table 6:** Component score coefficient matrix (coefficients by which variables are multiplied to obtain factor scores). The highest score coefficients in absolute value are marked in bold.

Variables	1st Principal Component (PC1)	2nd Principal Component (PC2)
Phenol content	−0.779	0.517
Flavonoid content	−**0.812**	0.447
Antioxidant_DPPH Assay	0.706	0.435
Antioxidant_H_2_O_2_ scavenging activity	**0.817**	−0.276
Antioxidant_NO scavenging activity	0.430	**0.692**
Antioxidant_Total antioxidant capacity	0.735	0.526

**Table 7:** IC_50_ of *R. cordifolia* extracts on viability of HeLa, ME-180 and HepG2 cells.

	Methanol Extract (mg/mL)	Ethanol Extract (mg/mL)	Aqueous Extract (mg/mL)	5-FU (μM)
HeLa	0.29 ± 0.23 ^a, c^	1.41 ± 0.37	0.51 ± 0.34 ^b^	34.73 ± 10.02
ME-180	1.68 ± 0.39 ^a^	2.37 ± 0.96 ^d^	1.78 ± 0.55 ^b^	13.68 ± 2.04
HepG2	0.38 ± 0.26	0.45 ± 0.07	0.57 ± 0.31	1.51 ± 0.38

For all the experiments, *n* = 3. ^a–d^ Column-wise values with different superscripts of this type indicate significant difference as determined by Student–Newman–Keuls method (*p* < 0.05); ^a^ between 5FU and methanol extract for same solvent; ^b–d^ for –PVPP for different solvents; ^b^ between 5-FU and aqueous; ^c^ between methanol and ethanol ^d^ and between 5-FU and ethanol.

**Table 8:** UPLC-UV-MS identification of major compounds from *Rubia cordifolia* L. root extract prepared in methanol. ID source: DBSearch.

No.	Name	Formula	Score	Mass	CAS	RT
1	Pseudopurpurin	C_15_H_8_O_7_	97.4	300.0275	476-41-5	9.442
2	Morindaparvin A	C_15_H_8_O_4_	84.38	252.0421	41621-32-3	10.821

## Data Availability

The data presented in this study are available within the article and supplementary material.

## Supplementary Materials

The following supporting information can be downloaded at: https://www.mdpi.com/article/10.3390/antiox11051006/s1. The following supporting information: Table S1. UPLC-UV-MS identification of possible compounds from *Rubia cordifolia* L. root extract prepared in methanol. ID source: DBSearch.

## Abbreviations

DPPH	2:2-diphenyl-1-picrylhydrazyl
PVPP	Polyvinylpolypyrrolidone
