# Supplementary material for: Phytochemical Characterization, Antioxidant and Anti-Proliferative Properties of Rubia cordifolia L. Extracts Prepared with Improved Extraction Conditions
Source: Antioxidants (Basel). 2022 May 20;11(5):1006. doi: 10.3390/antiox11051006 (PMC9137611; doi:10.3390/antiox11051006)
Supplement: Supplementary file 1 [file antioxidants-11-01006-s001.zip › antioxidants-1682768-supplementary.pdf]

Supplementary Data

Table S1. UPLC-UV-MS identification of possible compounds from *Rubia cordifolia* Linn. root extract prepared in methanol. ID source: DBSearch.

No.	Name	Formula	Score	Mass	CAS	RT
**1**	Ricinoleic acid	C18H34O3	99.67	298.2507	5323-95-5	15.677
**2**	Armepavine	C19H23NO3	99.54	313.168	524-20-9	16.405
**3**	Erysotrine	C19H23NO3	99.54	313.168	27740-43-8	16.405
**4**	Ethylmorphine	C19H23NO3	99.54	313.168	76-58-4	16.405
**5**	Laurifine	C19H23NO3	99.54	313.168	56261-28-0	16.405
**6**	Cryptotanshinone	C19H23O3	99.53	296.1415	35825-57-1	16.405
**7**	5-Deoxystrigol	C19H22O5	99.46	330.1468		11.167
**8**	Gibberellin A5	C19H22O5	99.46	330.1468		11.167
**9**	Gibberellin A51-catabolite	C19H22O5	99.46	330.1468		11.167
**10**	Gibberellin A7	C19H22O5	99.46	330.1468		11.167
**11**	Podolide	C19H22O5	99.46	330.1468	55786-36-2	11.167
**12**	Cyperine	C15H16O4	99.35	260.1051	33716-82-4	14.45
**13**	Hemigossypol	C15H16O4	99.35	260.1051		14.45
**14**	Homobaldrinal	C15H16O4	99.35	260.1051	67910-07-0	14.45
**15**	Linderane	C15H16O4	99.35	260.1051	13476-25-0	14.45
**16**	Pergillin	C15H16O4	99.35	260.1051	74798-20-2	14.45
**17**	Arachidic acid	C20H40O2	99.23	312.303	506-30-9	17.781
**18**	Phytanic acid	C20H40O2	99.23	312.303	14721-66-5	17.781
**19**	1,3,6,8-Naphthalenetetrol	C10H8O4	99.14	192.0424	18512-30-6	9.736
**20**	3,4-Dehydro-6-hydroxymellein	C10H8O4	99.14	192.0424		9.736
**21**	Acamelin	C10H8O4	99.14	192.0424	74161-27-6	9.736
**22**	Anemonin	C10H8O4	99.14	192.0424	508-44-1	9.736
**23**	Isoscopoletin	C10H8O4	99.14	192.0424	776-86-3	9.736
**24**	Naphthazarin	C10H8O4	99.14	192.0424	475-38-7	9.736
**25**	Scopoletin	C10H8O4	99.14	192.0424	92-61-5	9.736
**26**	Butyl octadecanoate	C22H44O2	99.03	340.3344	123-95-5	19.394
**27**	Docosanoic acid	C22H44O2	99.03	340.3344	112-85-6	19.394
**28**	2,3-Pentanedione	C5H8O2	98.96	100.0524	600-14-6	2.174
**29**	3-Methylbutyrolactone	C5H8O2	98.96	100.0524	1679-49-8	2.174
**30**	5-Aminopentanoic acid	C5H11NO2	98.96	117.079	660-88-8	2.174
**31**	L-Valine	C5H11NO2	98.96	117.079	72-18-4	2.174
**32**	Norvaline	C5H11NO2	98.96	117.079	760-78-1	2.174
**33**	Tiglic acid	C5H8O2	98.96	100.0524	80-59-1	2.174
**34**	Valine	C5H11NO2	98.96	117.079	516-06-3	2.174
**35**	Bombykol	C16H33O	98.58	238.2294		21.808
**36**	1-Acetoxypinoresinol	C22H24O8	98.51	416.1474	81426-14-4	10.817
**37**	Lophotoxin	C22H24O8	98.51	416.1474	78697-56-0	10.817

**38**	(S)-2-Methylbutanal	C5H10O	98.06	86.0729	1730-97-8	0.929
**39**	1-Penten-3-ol	C5H10O	98.06	86.0729	616-25-1	0.929
**40**	2-Methylbutanal	C5H10O	98.06	86.0729	96-17-3	0.929
**41**	Cyclopentanol	C5H10O	98.06	86.0729	96-41-3	0.929
**42**	pentan-2-one	C5H10O	98.06	86.0729		0.929
**43**	Stearic acid	C18H39O2	98.04	284.2718	57-11-4	16.174
**44**	Aspulvinone E	C17H15O5	98	296.0687	49637-60-7	11.387
**45**	Melicopine	C17H15NO5	98	313.0952	568-01-4	11.387
**46**	Butyl dodecanoate	C16H32O2	97.45	256.241	106-18-3	14.591
**47**	Octyl octanoate	C16H32O2	97.45	256.241	2306-88-9	14.591
**48**	Palmitic acid	C16H32O2	97.45	256.241	57-10-3	14.591
**49**	Decyl butanoate	C14H28O2	97.43	228.2089	01-09-54	12.986
**50**	Dicumarol	C19H15O6	97.43	336.0639	66-76-2	13.541
**51**	Myristic acid	C14H28O2	97.43	228.2089	544-63-8	12.986
**52**	Tetracenomycin D1	C19H15O6	97.43	336.0639		13.541
**53**	Protodioscin	C51H84O22	97.2	1048.547	55056-80-9	11.356
**54**	Trigonelloside C	C51H84O22	97.2	1048.547	60478-69-5	11.356
**55**	Cubebin	C20H20O6	97.08	356.1264	18423-69-3	10.838
**56**	Kievitone	C20H20O6	97.08	356.1264		10.838
**57**	Leachianone G	C20H20O6	97.08	356.1264		10.838
**58**	Plaunol B	C20H20O6	97.08	356.1264	69749-00-4	10.838
**59**	alpha-Irone	C14H22O	96.98	206.1665	79-69-6	1.221
**60**	1-Cyclohexene carboxylic acid	C7H10O2	96.55	126.0683	636-82-8	1.085
**61**	L-Thyronine	C15 H15 N O4	94.66	273.1012	1596-67-4	7.863
**62**	Angustine	C20H15N3O	94.5	313.1204	40041-96-1	11.167
**63**	Ochotensine	C21H21NO4	92.77	351.1473	4959-88-0	11.532
**64**	Caseadine	C20H23NO4	92.2	341.1632	34413-12-2	9.115
**65**	Isocorydine(+)	C20H23NO4	92.2	341.1632	475-67-2	9.115
**66**	(-)-Phaseollinisoflavan	C20H20O4	92.16	324.1367		9.115
**67**	Glabranin	C20H20O4	92.16	324.1367		9.115
**68**	Glabridin	C20H20O4	92.16	324.1367		9.115
**69**	Isobavachalcone	C20H20O4	92.16	324.1367		9.115
**70**	Moracin I	C20H20O4	92.16	324.1367	73338-88-2	9.115
**71**	Otobain	C20H20O4	92.16	324.1367	3738-01-0	9.115
**72**	Spathelia bischromene	C20H20O4	92.16	324.1367	34411-93-3	9.115
**73**	Cleomiscosin A	C20H18O8	91.42	386.102	76948-72-6	11.946
**74**	Daphneticin	C20H18O8	91.42	386.102	83327-22-4	11.946
**75**	Diferulic acid	C20H18O8	91.42	386.102	436-96-4	11.946
**76**	Irisflorentin	C20H18O8	91.42	386.102		11.946
**77**	(-)-8-Demethylmaritidine	C16H19NO3	86.45	273.1364		14.91
**78**	4'-O-Methylnorbelladine	C16H19NO3	86.45	273.1364		14.91

**79**	7,4'-Dihydroxy-8-methylflavan	C16H16O3	86.45	256.1098		14.91
**80**	Broussin	C16H16O3	86.45	256.1098		14.91
**81**	Lunacrine	C16H19NO3	86.45	273.1364	82-40-6	14.91
**82**	Orchinol	C16H16O3	86.45	256.1098	41060-20-2	14.91
**83**	Pterostilbene	C16H16O3	86.45	256.1098	537-42-8	14.91
**84**	Xenognosin A	C16H16O3	86.45	256.1098	76907-79-4	14.91
**85**	β-Erythroidine	C16H19NO3	86.45	273.1364	466-81-9	14.91
**86**	9(S)-HOTrE	C18H30O3	85.91	294.2193	89886-42-0	18.656
**87**	9-OxoODE	C18H30O3	85.91	294.2193	54232-59-6	18.656
**88**	Colneleic acid	C18H30O3	85.91	294.2193		18.656
**89**	Juvenile hormone I	C18H30O3	85.91	294.2193	13804-51-8	18.656
**90**	2-Hydroxypyridine	C5H5NO	85.9	95.0375	142-08-5	1.147
**91**	Alizarin	C14H8O4	85.55	240.042	72-48-0	10.912
**92**	Danthron	C14H8O4	85.55	240.042	117-10-2	10.912
**93**	7,4'-Dihydroxyflavone	C15H10O4	85.52	254.0581		12.019
**94**	Alizarin 2-methyl ether	C15H10O4	85.52	254.0581	08-11-03	12.019
**95**	Anhydroglycinol	C15H10O4	85.52	254.0581		12.019
**96**	Chrysin	C15H10O4	85.52	254.0581	480-40-0	12.019
**97**	Daidzein	C15H10O4	85.52	254.0581	486-66-8	12.019
**98**	Hispidol	C15H10O4	85.52	254.0581		12.019
**99**	Primetin	C15H10O4	85.52	254.0581		12.019
**100**	Rubiadin	C15H10O4	85.52	254.0581	117-02-2	12.019
**101**	(-)-Morphine	C17H19NO3	84.43	285.1363		15.27
**102**	(S)-Coclaurine	C17H19NO3	84.43	285.1363	486-39-5	15.27
**103**	Cherylline	C17H19NO3	84.43	285.1363	23367-61-5	15.27
**104**	Erysonine	C17H19NO3	84.43	285.1363	03-05-90	15.27
**105**	Morphine	C17H19NO3	84.43	285.1363	57-27-2	15.27
**106**	Narwedine	C17H19NO3	84.43	285.1363	510-77-0	15.27
**107**	Norcodeine	C17H19NO3	84.43	285.1363	467-15-2	15.27
**108**	Piperine	C17H19NO3	84.43	285.1363	94-62-2	15.27
**109**	11-Deoxylandomycinone	C19H14O5	83.96	322.0857		11.878
**110**	Tetrangomycin	C19 H14 O5	83.96	322.0857	08-08-51	11.878
**111**	Isosamidin	C21H22O7	82.58	386.1368	53023-18-0	10.853
**112**	Pteryxin	C21H22O7	82.58	386.1368	17944-23-9	10.853
**113**	Samidin	C21H22O7	82.58	386.1368	477-33-8	10.853
**114**	Chloroquine	C18H26ClN3	69.98	319.1817	54-05-7	9.115
